# The Role and Importance of Procalcitonin (PCT) Testing in Rational Antibiotic Prescribing and Antibiotic Stewardship Programs

**DOI:** 10.1002/iid3.70398

**Published:** 2026-04-26

**Authors:** Alireza Abdollahi, Aysan Nozheh, Kimia Mozahheb Yousefi, Mohammadreza Salehi, Zahra Modabber

**Affiliations:** ^1^ Department of Pathology, Cancer Institute, Imam Khomeini Hospital Complex Tehran University of Medical Sciences Tehran Iran; ^2^ Faculty of Medicine Tehran University of Medical Sciences Tehran Iran; ^3^ Antimicrobial Resistance Research Center, Institute of Immunology and Infectious Diseases Iran University of Medical Sciences Tehran Iran; ^4^ Faculty of Medicine Iran University of Medical Sciences Tehran Iran; ^5^ Research Center for Antibiotic Stewardship & Antimicrobial Resistance, Department of Infectious Diseases, Imam Khomeini Hospital Complex Tehran University of Medical Sciences Tehran Iran

**Keywords:** antibiotic resistance, antibiotic stewardship programs, antimicrobial therapy, ASPs, PCT, procalcitonin

## Abstract

**Introduction:**

Antibiotic resistance is a critical global health issue, primarily driven by inappropriate antibiotic prescribing. Identifying reliable biomarkers to guide antibiotic use is essential to combat this challenge. Procalcitonin (PCT) testing has emerged as a promising tool for differentiating bacterial infections from non‐bacterial conditions and supporting antibiotic stewardship programs. This literature review evaluates evidence from randomized controlled trials, observational studies, and real‐world clinical data to assess the clinical utility of PCT testing in guiding antibiotic prescribing decisions.

**Results:**

Across randomized controlled trials and real‐world studies, PCT‐guided algorithms typically yielded reductions in antibiotic duration of approximately 0.9 to 3 days in large adult cohorts, with some smaller or source‐specific trials reporting reductions of up to 6 days. Relative decreases in antibiotic exposure varied widely across studies (ranging from approximately 8% to > 50%). Importantly, these reductions were achieved without increases in short‐term or 28‐day mortality, while hospital length of stay remained largely unchanged. Studies generally indicate that PCT‐guided strategies are cost‐effective, primarily driven by reduced antibiotic use. However, challenges such as interpretive difficulties, false‐positive and negative results, and implementation barriers remain.

**Conclusion:**

Incorporating PCT testing into antibiotic stewardship strategies offers a promising approach to enhance prescribing accuracy, reduce antibiotic resistance risks, and improve patient care quality. Future research should focus on refining clinical guidelines, expanding applicability across diverse patient populations, and addressing practical challenges to facilitate widespread adoption of PCT testing in routine clinical settings.

## 1 Introduction

Antibiotic resistance is a major global health issue, posing serious challenges to public health worldwide. The inappropriate or excessive use of antibiotics contributes significantly to the development and spread of resistant organisms, leading to treatment failures, prolonged hospitalizations, higher healthcare costs, and increased morbidity and mortality rates. Addressing antibiotic resistance requires urgent action and coordinated strategies at multiple healthcare levels [1, 2].

Antibiotic stewardship programs (ASPs) have emerged as crucial interventions to ensure antibiotics are used responsibly and effectively. The main objective of ASPs is to optimize antibiotic prescribing by promoting appropriate selection, dosing, duration, and administration routes. Effective implementation of ASPs reduces unnecessary antibiotic exposure, minimizes the emergence of resistance, and improves clinical outcomes for patients [3, 4].

Biomarkers play a critical role in diagnosing infections and guiding therapeutic decisions. Accurate biomarkers assist healthcare providers in differentiating bacterial infections from other inflammatory conditions, facilitating timely and appropriate antibiotic use. Among these biomarkers, procalcitonin (PCT) has gained significant attention due to its specificity and reliability in identifying bacterial infections and managing antibiotic therapy effectively [5, 6, 7].

The primary aim of this review is to critically examine and clarify the role and clinical value of PCT testing in guiding rational antibiotic prescribing practices and enhancing ASPs. With growing concerns surrounding antibiotic resistance worldwide, healthcare systems require evidence‐based approaches to optimize antibiotic use [8, 9]. Biomarkers such as PCT have emerged as promising tools that could improve clinical decision‐making, reduce unnecessary antibiotic exposure, and ultimately limit the spread of resistant pathogens. Despite increasing recognition, variations and uncertainties in clinical guidelines still exist regarding the precise role of PCT testing [10, 11].

This review aims to consolidate current evidence, identify gaps in the existing literature, and provide clear recommendations that could help inform future practice and policy decisions in antibiotic stewardship.

## 2 PCT as a Biomarker

### 2.1 Biological Role and Kinetics of PCT

PCT is a protein precursor of the hormone calcitonin, primarily produced under normal physiological conditions in the parafollicular C‐cells of the thyroid gland, with minimal concentrations detectable in the blood of healthy individuals. Unlike calcitonin, which is involved in calcium homeostasis, PCT itself has no known physiological function at baseline concentrations [12, 13, 14]. However, in response to systemic bacterial infections or significant inflammation caused by microbial pathogens, extra‐thyroidal tissues (including the lungs, liver, kidneys, adipose tissue, and gastrointestinal tract) begin synthesizing and releasing substantial amounts of PCT. This process is triggered mainly by pro‐inflammatory cytokines such as interleukin‐6 (IL‐6), tumor necrosis factor‐alpha (TNF‐α), and interleukin‐1 beta (IL‐1β), stimulated by bacterial toxins, notably endotoxins. This rapid and significant increase in PCT levels distinguishes bacterial infections from viral infections or non‐infectious inflammatory states, providing clinicians with a valuable diagnostic biomarker that can accurately identify bacterial infections and thereby guide clinical decision‐making effectively [9, 10, 11].

Clinically, PCT demonstrates rapid kinetics in response to bacterial infections, making it particularly useful in acute clinical settings. Following exposure to bacterial pathogens, PCT concentrations in blood serum begin to rise noticeably within 2 to 4 h, reaching peak values typically within 12 to 24 h after infection onset. Its serum levels correlate closely with the severity of bacterial infection, making it not only diagnostic but also prognostic. Once appropriate antibiotic therapy is initiated, and the bacterial burden is reduced, PCT levels decline steadily, typically halving every 24 to 36 h [12, 13, 14, 15]. This predictable decrease in PCT concentrations provides a measurable indicator of treatment effectiveness, facilitating decisions regarding antibiotic duration, dosage adjustments, or cessation. Moreover, persistently elevated PCT levels despite antibiotic treatment may indicate inadequate therapy, complications, or secondary infections, thereby prompting further clinical interventions. Understanding these kinetics enables clinicians to optimize antibiotic management, improve patient outcomes, reduce antibiotic exposure duration, and minimize the emergence of antimicrobial resistance (AMR) [16, 17, 18, 19].

### 2.2 Comparative Analysis of PCT Versus Other Biomarkers

PCT has emerged as a promising biomarker when compared to traditional inflammatory markers such as C‐reactive protein (CRP), white blood cell count (WBC), erythrocyte sedimentation rate (ESR), and selected cytokines (e.g., IL‐6, IL‐8). While CRP and WBC are widely used as in evaluating inflammation or suspected infection, both lack specificity because their levels may rise in viral infections, autoimmune disorders, and other non‐infectious inflammatory conditions [16, 17, 18, 19].

Comparative studies demonstrate that the diagnostic performance of PCT relative to these markers varies substantially according to clinical syndrome and disease severity. For sepsis, pooled estimates from a recent meta‐analysis report PCT sensitivity of approximately 0.70 and specificity of 0.67 (AUC 0.74). In comparison, CRP demonstrates comparable sensitivity (0.72) but markedly lower specificity (0.55) in critically ill populations, limiting its ability to reliably distinguish bacterial infection from non‐infectious inflammation (AUC 0.67) [20]. Similarly, ESR and older cytokine assays often have limited utility due to lower specificity, slower kinetics, and the need for specialized assays.

Emerging biomarkers such as presepsin and IL‐6 have shown comparable diagnostic performance. A recent meta‐analysis reported a pooled sensitivity for presepsin of 0.82 and specificity of 0.78, concluding that its diagnostic performance is comparable to PCT [21]. Similarly, regarding IL‐6, recent data suggest that monitoring the dynamic change in IL‐6 levels over the first 3 days of sepsis provides reliable mortality prediction, particularly when combined with PCT and presepsin [22].

Despite these nuances, PCT demonstrates high specificity for bacterial infections and exhibits rapid, predictable kinetics, making it valuable for distinguishing bacterial from viral or inflammatory etiologies [23, 24, 25, 26, 27, 28]. These characteristics support more targeted antibiotic prescribing, with studies showing improved clinical outcomes, reduced antibiotic exposure, and lower healthcare costs in PCT‐guided algorithms [26, 27, 28, 29, 30].

However, limitations exist. These data underscore that no single biomarker provides uniformly high sensitivity and specificity across all syndromes. False‐positive elevations may occur in severe trauma, major surgery, or burns, and low PCT levels cannot fully exclude localized or mild bacterial infections [31, 32, 33]. Furthermore, cost and limited availability can restrict its use in resource‐limited settings. Accordingly, current evidence supports the use of PCT and other biomarkers as adjunctive tools whose interpretation must be contextualized to the underlying disease process, clinical setting, and pretest probability, rather than as standalone diagnostic tests [34, 35, 36].

## 3 Clinical Applications of PCT Testing

### 3.1 Diagnosis of Bacterial Infections

PCT testing plays an important role in distinguishing bacterial infections from viral or non‐infectious inflammatory conditions due to its infection‐specific expression pattern [37, 38, 39]. Elevated PCT levels strongly suggest a bacterial etiology and support timely initiation of antimicrobial therapy, whereas low levels indicate a lower probability of bacterial infection, helping reduce unnecessary antibiotic use [39, 40].

PCT is especially valuable in conditions such as sepsis and respiratory tract infections. In sepsis, PCT correlates with severity and assists in early diagnosis, risk stratification, and monitoring treatment response [41, 42]. Crucially, the most robust evidence for PCT‐guided antibiotic stewardship derives from studies of undifferentiated sepsis in the intensive care unit (ICU), where the infection source is often initially unknown. In this setting, major trials such as ADAPT‐Sepsis demonstrate that serial PCT monitoring facilitates the safe de‐escalation of empiric antibiotics without increasing mortality [43]. However, diagnostic performance varies across source‐specific syndromes. For example, PCT diagnostic value is notably lower in intra‐abdominal infections due to confounding factors like pancreatitis [44], compared to its established high performance in pulmonary sources. In respiratory infections like community‐acquired pneumonia, PCT‐guided algorithms effectively differentiate bacterial from non‐bacterial etiologies, reducing unnecessary antibiotic exposure and improving patient outcomes [45, 46, 47]. Consequently, PCT results must be interpreted within the broader clinical context rather than as a standalone diagnostic tool.

### 3.2 Guiding Antibiotic Therapy

PCT serves as a widely utilized biomarker in antimicrobial stewardship, guiding clinicians in the initiation, escalation, or de‐escalation of therapy based on serum levels. While elevated PCT provides a strong indication for initiating antibiotics in patients with suspected bacterial infections (particularly in sepsis and lower respiratory tract infections), its utility extends to the dynamic monitoring of therapeutic response [42, 45, 46]. Specifically, stable or declining PCT levels support evidence‐based decisions to de‐escalate or discontinue antibiotics, thereby avoiding unnecessary prolonged exposure [46, 47, 48]. Conversely, when levels remain persistently high or fail to decrease, clinicians are prompted to escalate therapy or investigate complications, resistance, or secondary infections. This biomarker‐driven approach refines therapeutic decision‐making, ultimately optimizing patient care [48, 49, 50].

The integration of these PCT‐guided protocols into clinical practice frequently demonstrates a reduction in the duration of antibiotic therapy without compromising clinical outcomes. By providing objective criteria based on dynamic changes in serum concentrations, clinicians can confidently shorten treatment courses [47, 48, 49], minimizing antibiotic exposure and decreasing the risk of adverse drug reactions and the development of AMR [49, 50, 51]. Furthermore, clinical evidence indicates that patients managed according to PCT protocols frequently experience fewer healthcare‐associated infections, shorter hospitalizations, and improved recovery rates. Consequently, PCT implementation enhances clinical efficacy and safety while lowering healthcare costs, aligning effectively with current antimicrobial stewardship strategies [52, 53, 54].

However, literature regarding PCT monitoring reveals mixed results, and efficacy is not uniform across all clinical scenarios. While PCT usage is suggested for tailoring antibiotic therapy, its utility in other sources, such as intra‐abdominal infections, appears limited. An observational study indicated that standard PCT thresholds failed to accurately predict initial treatment response in perioperative intra‐abdominal infections presenting with septic shock. Specifically, a PCT decrease to 0.5 ng/mL lacked sensitivity, as 50% of the patients who remained above this level still successfully responded to treatment [55].

Additionally, the implementation of PCT testing is challenged by the lack of a universal consensus on algorithms. Diagnostic protocols vary widely, ranging from absolute discontinuation thresholds (e.g., < 0.25 µg/L or < 0.5 µg/L) to relative criteria (e.g., a decrease of > 80%), complicating the standardization of guidelines. This is particularly relevant in the ICU, where chronic kidney disease and renal replacement therapy may alter baseline PCT levels, potentially complicating diagnosis [56]. Patient demographics introduce further nuance, rendering static adult‐derived references inadequate for pediatric populations. Research indicates that physiological PCT baselines differ significantly by gestational maturity. Specifically, preterm infants exhibit naturally higher peak concentrations and require up to 9 weeks to reach adult reference levels, whereas term infants normalize within 5 days [57]. Consequently, diagnostic cut‐offs must be age‐adjusted. Furthermore, because PCT kinetics correlate with pediatric organ dysfunction scores [58], clinical algorithms must rely on age‐specific percentile curves and dynamic monitoring rather than a “one‐size‐fits‐all” approach.

## 4 Impact of PCT on Antibiotic Stewardship Programs

### 4.1 Overview of Antibiotic Stewardship

ASPs are systematic initiatives designed to encourage the responsible use of antibiotics, aiming primarily to optimize patient outcomes while minimizing unintended consequences such as antibiotic resistance, adverse drug events, and healthcare costs. The core goals of ASPs involve ensuring appropriate antibiotic selection, correct dosing, suitable administration routes, and precise treatment durations [54, 59, 60]. Fundamental principles include promoting evidence‐based prescribing, continuous clinical assessment, education and training of healthcare professionals, surveillance of antibiotic usage, and regular feedback on prescribing practices. Key stewardship practices incorporate formulary restrictions, guidelines for empirical and targeted therapies, antibiotic “time‐outs,” therapeutic audits, and rapid diagnostic testing to guide treatment decisions [54, 59, 60].

Despite substantial progress, ASPs still face significant challenges and barriers to widespread implementation. Key issues include inadequate resources, limited availability of trained personnel specializing in infectious diseases or antimicrobial stewardship, and insufficient administrative or institutional support. Resistance from healthcare providers due to concerns about clinical autonomy or perceived risks of undertreatment can also impede the effective implementation of ASPs. Additionally, variability in compliance with established guidelines, difficulties in accurate and timely diagnosis of infections, and inconsistent monitoring of antibiotic use further complicate stewardship efforts [60, 61, 62]. These obstacles highlight the importance of developing more accessible, effective, and evidence‐based stewardship interventions, such as incorporating biomarkers like PCT into antibiotic decision‐making protocols [61, 62, 63].

### 4.2 Integration of PCT in Stewardship Practices

PCT testing has been successfully integrated into antibiotic stewardship initiatives in various clinical environments, including hospital wards, ICUs, and emergency departments. Clinical studies and hospital‐based quality improvement programs have demonstrated the effective implementation of PCT‐guided algorithms, resulting in improved prescribing behaviors and adherence to antimicrobial stewardship guidelines [63, 64, 65]. For instance, hospitals employing structured PCT protocols for respiratory tract infections and sepsis management have reported decreases in antibiotic prescription rates, shorter durations of therapy, and reduced hospitalization times. These outcomes illustrate how PCT testing can support clinical decision‐making by providing clear, evidence‐based guidelines for initiating and discontinuing antibiotics, ultimately facilitating more precise and responsible antibiotic use [65, 66].

The inclusion of PCT testing within antibiotic stewardship practices has substantially influenced antibiotic usage patterns. Clinical evidence indicates that adopting PCT‐driven treatment decisions effectively reduces unnecessary antibiotic prescriptions, lowers overall antibiotic consumption, and shortens the length of antibiotic treatment courses [65, 66]. Consequently, these reductions contribute to a lower selection pressure for resistant organisms, potentially decreasing the prevalence and spread of antibiotic resistance within healthcare facilities. Several hospital‐based studies have correlated the use of PCT guidance with measurable decreases in multidrug‐resistant infections, reflecting the broader benefit of biomarker‐assisted antibiotic management strategies in combating AMR at institutional and community levels [66, 67].

### 4.3 Cost‐Effectiveness and Economic Implications

The integration of PCT testing to guide antibiotic prescribing offers notable economic advantages for healthcare systems. By facilitating accurate diagnosis and timely therapy adjustments, PCT‐driven protocols can reduce unnecessary antibiotic exposure. Additionally, limiting antibiotic use minimizes associated complications (such as antibiotic‐related side effects, adverse drug reactions, and healthcare‐associated infections), further reducing medical costs and alleviating financial strain on health services [67, 68, 69].

Numerous cost‐effectiveness analyses have evaluated the economic impact of implementing PCT‐guided antibiotic treatment protocols across various clinical contexts. These studies generally indicate that PCT testing is cost‐effective due to savings derived primarily from shorter antibiotic courses and decreased antibiotic consumption. For example, analyses focusing on respiratory infections and sepsis consistently report cost reductions compared to traditional antibiotic prescribing practices, highlighting notable savings per patient or per hospital admission [7, 11, 13]. Despite initial costs associated with introducing PCT assays, these studies collectively suggest that the long‐term economic benefits outweigh upfront expenditures.

Recent health economic evaluations have further quantified these financial impacts, providing granular data on sepsis management. A 2025 cost‐effectiveness analysis utilizing meta‐analyzed data demonstrated that PCT‐guided antibiotic discontinuation represents a high‐value intervention, achieving a cost per quality‐adjusted life year gained of €2384, well below standard European thresholds. Importantly, when the analysis was restricted strictly to antibiotic use and testing costs, the intervention incurred only modest incremental costs (≤€110 per patient) while delivering improved stewardship [70].

However, budget impact analyses suggest that the most substantial savings arise when downstream effects, such as reduced ICU length of stay, are incorporated. A recent European model reported that PCT‐guided stewardship was associated with a reduction of approximately 1.5 ICU days per patient, yielding total net cost savings of approximately €1405 per patient [64]. Collectively, while direct antibiotic cost reductions are modest, the inclusion of reduced ICU and hospital occupancy indicates a tiered economic benefit. These findings reinforce the financial rationale for incorporating PCT‐based decision‐making into ASP, validating its value beyond clinical outcomes alone.

## 5 Evidence From Clinical Studies and Trials

Several randomized controlled trials (RCTs) have extensively investigated the utility of PCT testing in antibiotic prescribing practices across different clinical settings. Prominent trials have included settings such as ICUs, emergency departments, and general medical wards, particularly focusing on respiratory tract infections, sepsis, and other bacterial illnesses. Findings from these key trials consistently demonstrated that using PCT‐guided algorithms reduced antibiotic exposure, shortened treatment durations, and decreased antibiotic prescription rates without compromising patient safety or clinical outcomes [8, 9, 10].

Table 1 summarizes key RCTs evaluating PCT‐guided antibiotic stewardship across different clinical settings, highlighting comparative effects on antibiotic duration, length of stay, mortality, ICU admission, and economic outcomes. Notably, the landmark ProHOSP trial, which utilized a PCT cut‐off of < 0.25 µg/L to encourage antibiotic discontinuation in patients with lower respiratory tract infections (LRTIs), reported a significant reduction in antibiotic exposure from a mean of 8.7 days in the control group to 5.7 days in the PCT group, representing a 34.5% reduction in total antibiotic use [73]. Similarly, the PRORATA trial in the ICU setting applied a protocol recommending cessation when PCT levels dropped below 0.5 µg/L or decreased by ≥ 80% from the peak value. This approach resulted in 2.7 more antibiotic‐free days over a 28‐day period compared to standard care, without impacting 60‐day mortality [72].

**TABLE 1:** Comparative summary of key outcomes from randomized controlled trials (RCTs) on procalcitonin‐guided antibiotic stewardship.

Study (year)	Population	Infection	Comparator	Antibiotic duration (days)	Hospital length of stay	Mortality
ADAPT‐Sepsis (2025) [43]	Adults with suspected sepsis in 41 UK ICUs (*n* = 2760)	Suspected sepsis	Standard care vs. daily PCT algorithm	10.7 (standard care) vs. 9.8 (PCT) (*p* = 0.01)	Not Reported (ICU trial)	19.4% (standard care) vs. 20.9% (PCT)
SAPS (2016) [71]	Critically ill adults in 15 Netherlands ICUs (*n* = 1575)	Various	Standard care vs. PCT‐guided cessation	7 (standard care) vs. 5 (PCT) (*p* < 0.0001)	Not Reported (ICU trial)	25% (standard care) vs. 20% (PCT)
PRORATA (2010) [72]	Adults in 6 France ICUs (*n* = 621)	Suspected bacterial infections	Guideline‐based care vs. PCT‐guided care	Antibiotic free days:11.6 (control) vs. 14.3 (PCT)	Not Reported (ICU trial)	20.4% (control) vs. 21.2% (PCT)
PROHOSP (2009) [73]	Adults with lower respiratory tract infection in six Swiss EDs (*n* = 1359)	CAP, COPD exacerbation, acute bronchitis	Standard guidelines vs. PCT algorithm	8.7 (standard care) vs. 5.7 (PCT)	Similar in both groups	Adverse‐outcome rate:18.9% (control) vs. 15.4% (PCT)
PROGRESS (2025) [74]	Adults with sepsis in Greece (*n* = 157)	Sepsis	Standard duration vs. Early PCT‐guided cessation	11 (standard care) vs. 5 (PCT) (*p* < 0.001)	Not Reported	Not Reported
BATCH (2025) [75]	Hospitalized children with bacterial infections in the UK (*n* = 1949)	Mixed	Standard care vs. PCT algorithm	99.7 h (standard care) vs. 96 h (PCT)	Similar in both groups	Adverse‐outcome rate: 9% (standard care) vs. 9% (PCT)
ProPAED (2013) [76]	Children with lower respiratory tract infection in Switzerland (*n* = 337)	Lower respiratory tract infection	Standard guidelines vs. PCT algorithm	6.3 (standard care) vs. 4.5 (PCT) (*p* = 0.03)	Similar in both groups	Similar serious adverse events (SAE) and complications
ProPICU (2021) [77]	Children in a US PICU (*n* = 270)	Suspected infection	Standard care vs. PCT algorithm	7.6 (standard care) vs. 6.6 (PCT) (*p* = 0.37)	7 days (standard care) vs. 6 days (PCT) (No difference)	4 (standard care) vs. 3 (PCT) (No difference)
CAP‐China PCT‐AECOP (2025) [78]	Hospitalized adults with AECOPD across six centers in China (*n* = 455)	Acute exacerbation of COPD	GOLD guidelines vs. PCT‐guided care	4.86 (GOLD) vs. 2.63 (PCT) (*p* < 0.0001)	8.08 (GOLD) vs. 8.46 (PCT) (*p* = 0.27)	2 (GOLD) vs. 0 (PCT) (*p* = 0.25)

The evidence generated by these RCTs is generally considered robust due to rigorous study designs, clear methodological standards, and strong statistical power. Most high‐quality RCTs incorporated clear randomization procedures, defined clinical endpoints, and adequate patient follow‐up, enhancing the reliability and validity of findings. However, certain limitations, such as potential selection bias, variability in study populations, differences in clinical settings, or inconsistent adherence to PCT protocols, have been acknowledged and assessed [18, 23, 24]. Overall, systematic reviews and meta‐analyses affirm that the current evidence supporting PCT‐based antibiotic stewardship is of high quality, providing strong justification for integrating PCT‐guided approaches into clinical practice to optimize antibiotic management [26, 27, 29]. Notably, a meta‐analysis encompassing 18 trials and 3174 patients with LRTIs evaluated the ability of PCT to distinguish between pathogen types. The study found that at a cutoff of 0.5 µg/L, PCT demonstrated moderate diagnostic accuracy, showing a pooled specificity of 0.80 (95% CI: 0.65–0.90) for differentiating bacterial from viral infections, and a specificity of 0.81 (95% CI: 0.61–0.92) for distinguishing typical from atypical pathogens [79].

Observational studies conducted in real‐world clinical settings complement RCTs by providing additional insights into the practical implementation of PCT testing for antibiotic stewardship. To improve the global applicability of these findings, recent studies from Asia, Africa, and Latin America have also evaluated the role of PCT‐guided antibiotic stewardship across diverse healthcare settings. Recent studies from Asia indicate that PCT algorithms can safely shorten antibiotic courses and improve stewardship outside Europe and the US. For example, in India, a before‐after ICU study (*n* = 300) found that routine PCT monitoring significantly increased antibiotic de‐escalation and cut ICU stay by approximately 2 days. The authors concluded that PCT guidance enabled earlier discontinuation of antibiotics and shorter ICU stays [80]. Moreover, in Indian children with sepsis and LRI, a PCT‐based protocol significantly reduced antibiotic durations and hospital stay without excess morbidity [29]. A Malaysian study in ventilator‐associated pneumonia also showed that adding point‐of‐care PCT testing reduced mean antibiotic use (*p* < 0.05) [81]. By contrast, a recent Jordanian ICU trial in cancer patients found no difference in antibiotic duration between PCT‐guided and standard care [82]. Data from Africa and Latin America are still limited. In Ethiopia, point‐of‐care PCT assays had excellent diagnostic accuracy (AUC 0.91–0.99) for detecting bacterial infections against a composite reference standard, suggesting that PCT could help avoid unnecessary antibiotics in resource‐poor settings [83]. In Latin America, evidence is sparse. One Brazilian ICU trial reported that PCT‐guided stopping rules produced antibiotic durations similar to CRP‐guided care [84]. Taken together, while current data are encouraging, more prospective trials in African and Latin American populations are needed to confirm these clinical benefits.

These studies, carried out in diverse healthcare environments such as hospitals, emergency departments, and outpatient clinics, have consistently demonstrated that PCT‐guided protocols effectively decrease antibiotic prescribing rates, reduce unnecessary antibiotic exposure, and shorten treatment durations [31, 32, 33]. Real‐world evidence also indicates improved adherence to antibiotic stewardship guidelines when clinicians incorporate PCT results into their clinical decision‐making processes. Consequently, observational data reinforce the feasibility and practical benefits of using PCT testing as part of routine antibiotic management strategies [34, 35].

Despite their practical relevance, observational studies have inherent limitations and potential biases that must be considered when interpreting their results. Common limitations include selection bias, which occurs when study populations differ systematically from the general patient population, potentially affecting generalizability. Additionally, confounding factors (such as varying clinician experience, inconsistent adherence to protocols, and differences in healthcare settings) may influence outcomes independently of PCT testing [36, 37]. Observational studies also lack randomization, which reduces their ability to establish clear cause‐and‐effect relationships. Therefore, although observational evidence is valuable for demonstrating real‐world applicability, results must be interpreted cautiously and ideally complemented by RCT data to support robust clinical recommendations [33, 35, 37].

## 6 Challenges and Limitations

Interpreting PCT levels in clinical practice presents certain challenges. Although guidelines provide thresholds for clinical decisions, the interpretation of PCT values can be complicated due to variations in individual patient responses, the severity of infection, and the timing of sample collection. Additionally, PCT levels may be influenced by factors such as underlying chronic diseases, immunosuppression, or previous antibiotic treatments, potentially leading to uncertainties or inconsistent clinical interpretations [36, 38, 39]. This complexity underscores the necessity for clinical judgment to supplement biomarker measurements, emphasizing the importance of integrating PCT testing within broader clinical assessments [49, 53, 59].

PCT testing, despite its specificity, is not immune to false‐positive or false‐negative results. Elevated PCT levels can occasionally occur without bacterial infections, such as in conditions involving severe trauma, major surgery, burns, acute pancreatitis, or cardiogenic shock, potentially leading to unnecessary antibiotic administration [51, 61, 62]. Conversely, low or negative PCT levels do not definitively rule out mild or localized bacterial infections, possibly causing delays or missed opportunities in initiating appropriate antibiotic therapy. Such inaccuracies have important clinical implications, highlighting the need for clinicians to carefully correlate PCT results with clinical context and additional diagnostic investigations to avoid mismanagement [61, 64, 67].

Several barriers may hinder the widespread implementation of PCT testing in routine clinical practice. These barriers include the initial cost and availability of testing, particularly in resource‐constrained healthcare settings. Importantly, the adoption of PCT‐guided antibiotic stewardship in low‐ and middle‐income countries (LMICs) faces substantial structural and economic barriers that limit scalability and equitable implementation. Although LMICs bear a disproportionate share of the global AMR burden, with the majority of AMR‐attributable mortality occurring in these settings [85], access to advanced diagnostic biomarkers such as PCT remains limited due to high per‐test costs, reagent supply constraints, and inadequate laboratory infrastructure and personnel [86, 87]. Furthermore, much of the evidence supporting PCT‐guided stewardship has been generated in high‐income healthcare settings, raising concerns about the generalizability, affordability, and feasibility of these strategies in LMIC health systems.

The introduction of new diagnostic tools such as PCT requires clinician education, training, and institutional support, which can be resource‐intensive and challenging to achieve. Resistance to adopting new diagnostic algorithms due to traditional prescribing practices, clinical inertia, or skepticism about new biomarkers also contributes to implementation challenges [19, 26, 59]. Crucially, the actual role of PCT in guiding treatment is far from standardized. Usage remains highly institution‐specific, with evidence showing variability in protocol adherence and utilization rates even across different units within the same hospital. Addressing these barriers requires systematic approaches, such as educational initiatives, supportive policies, and demonstrating clear clinical and economic benefits to promote acceptance and integration of PCT‐guided protocols into standard clinical practice.

## 7 Recommendations and Future Directions

PCT testing should be prioritized in clinical scenarios where distinguishing bacterial infections from non‐bacterial inflammatory or viral conditions is particularly critical. Key recommended scenarios include suspected sepsis or septic shock, LRTIs like pneumonia or acute exacerbations of chronic obstructive pulmonary disease (COPD), febrile neutropenia in immunocompromised patients, postoperative infections, and meningitis. Patient populations who would benefit from routine PCT monitoring include critically ill patients, hospitalized individuals with unclear infection diagnoses, and those at increased risk for antibiotic‐related complications or resistance development. Incorporating PCT testing in these contexts supports more accurate and timely antibiotic treatment decisions, ultimately enhancing patient care [3, 9, 24, 28].

To effectively integrate PCT testing into ASPs, healthcare institutions should adopt structured implementation strategies. These strategies include establishing clear institutional guidelines and algorithms specifying threshold levels for initiating and discontinuing antibiotics based on PCT results. Regular training sessions and continuous educational initiatives for healthcare providers are crucial to enhance understanding, promote acceptance, and ensure consistent application of PCT‐based protocols. Furthermore, ongoing monitoring, auditing, and feedback mechanisms are essential to evaluate protocol adherence, clinical outcomes, and economic impacts, fostering continuous quality improvement. Strong institutional support, adequate resource allocation, and interdisciplinary collaboration among clinicians, microbiologists, pharmacists, and stewardship teams are also vital to successfully embed PCT testing into routine clinical practice.

Although substantial evidence supports the clinical utility of PCT, several research gaps remain that require further investigation. Future research should focus on optimizing the precise threshold levels of PCT in various clinical contexts, particularly in pediatric and immunocompromised populations, where existing data remain limited. Additional studies evaluating the impact of PCT testing on long‐term antibiotic resistance trends and healthcare‐associated infection rates are essential. Research exploring the cost‐effectiveness of PCT testing in different resource settings, including LMICs, is also needed to support global implementation. Moreover, investigation into combining PCT with other emerging biomarkers or diagnostic tools may enhance diagnostic accuracy and clinical outcomes, further advancing the effectiveness of antimicrobial stewardship practices.

## 8 Conclusion

This review underscores the potential utility of PCT testing as a valuable adjunct for informing antibiotic prescribing and supporting ASPs. Evidence from clinical studies suggests that, when applied within structured protocols, PCT‐based approaches can assist in differentiating bacterial from non‐bacterial conditions, thereby potentially reducing unnecessary antibiotic exposure and shortening treatment durations. While integrating PCT into clinical decision‐making offers opportunities for economic advantages and resistance control, its impact is currently limited by variability in usage and a lack of standardization across institutions. Future strategies must therefore focus on harmonizing implementation guidelines and addressing context‐specific barriers to ensure that PCT testing can be consistently leveraged to improve public health outcomes.

## Author Contributions

**Aysan Nozheh:** investigation, resources, validation, writing – review and editing, writing – original draft. **Mohammadreza Salehi:** data curation, resources, validation, writing – review and editing. **Zahra Modabber:** investigation, data curation, writing – original draft, formal analysis, software, writing – review and editing. **Alireza Abdollahi:** conceptualization, methodology, project administration, supervision, writing – original draft. **Kimia Mozahheb Yousefi:** investigation, data curation, resources, validation, writing – review and editing, visualization.

## Funding

The authors have nothing to report.

## Conflicts of Interest

The authors declare no conflicts of interest.
